# Investigation of Anthrax Cases in North-East China, 2010-2014

**DOI:** 10.1371/journal.pone.0135777

**Published:** 2015-08-26

**Authors:** Wei Zhou, Yang Sun, Lingwei Zhu, Bo Zhou, Jun Liu, Xue Ji, Xiaofeng Wang, Nan Wang, Guibo Gu, Shuzhang Feng, Jun Qian, Xuejun Guo

**Affiliations:** 1 Institute of Military Veterinary, AMMS, Key Laboratory of Jilin Province for Zoonosis Prevention and Control, Changchun, Jilin, China; 2 Animal Diseases Control and Prevention Centre of Jilin Province, Jilin, China; 3 Animal Diseases Control and Prevention Centre of Liaoning Province, Liaoning, China; ContraFect Corporation, UNITED STATES

## Abstract

We determined the genotypes of seven *Bacillus anthracis* strains that were recovered from nine anthrax outbreaks in North-East China from 2010 to 2014, and two approved vaccine strains that are currently in use in China. The causes of these cases were partly due to local farmers being unaware of the presence of anthrax, and butchers with open wounds having direct contact with anthrax-contaminated meat products. The genotype of five of the seven recovered strains was A.Br.001/002 sub-lineage, which was concordant with previously published research. The remaining two cases belongs to the A.Br.Ames sub-lineage. Both of these strains displayed an identical SNR pattern, which was the first time that this genotype was identified in North-East China. Strengthening education in remote villages of rural China is an important activity aimed at fostering attempts to prevent and control anthrax. The genotype of the vaccine strain Anthrax Spore Vaccine No.II was A.Br.008/009 and A.Br.001/002 for the vaccine strain Anthrax Spore Vaccine Non-capsulated. Further studies of their characteristics are clearly warranted.

## Introduction

*Bacillus anthracis* is a notorious pathogen that is responsible for anthrax outbreaks, a disease that is potentially lethal to humans. Anthrax outbreaks can also cause the death of wild animals and livestock. Moreover, *Bacillus anthracis* can be adapted for use as a biological warfare agent. It not only threatens human and animal life, but also might provoke social unrest and panic [1–3].

Anthrax is a globally widespread zoonosis. Sporadic cases of anthrax were reported occasionally all over the world [4–8]. Until now, no country has yet to announce the eradication of anthrax. Part of the reason for this is that the bacteria can persist in the environment as a dormant and highly stable spore that is capable of enduring extreme conditions. Furthermore, *Bacillus anthracis* spore can germinate and recover its ability to infect animals or individuals once it finds itself in a suitable environment [9]. In addition, heavy rains and flooding, especially in the summer months, bring spores that are normally found under the soil to the surface. With these conditions, the spores are able to pool in low-lying areas, and following evaporation of the surface water, spores might attach to the shoots of growing plants or they might concentrate in the soil around the roots. Once herbivorous animals graze, there is an opportunity for these animals to be readily infected [10].

Since the dormant spores of *B*.*anthracis* lack active metabolism, *B*.*anthracis* evolves slowly and has limited genetic variability in the dormant form. However, many groups have paid increased attention to the presence and risks of anthrax, especially after the events of anthrax mail attacks that took place in the U.S. following the terrorist attacks of September 11, 2001 [11].

Based on the information gained from whole genome sequences, precise methods that have included Multi-Locus Variable-number tandem-repeat Analysis (MLVA), canonical Single Nucleotide Polymorphism (canSNP) and Single-Nucleotide Repeat analysis (SNR) are widely used to discover the molecular genetic characters of *B*. *anthracis*. Using eight VNTRs (MLVA8), Keim *et al*.[12] first subtyped a global collection of over 400 *B*. *anthracis* strains into 89 genotypes that defined two major clonal lineages (referred to as A and B). Following that, and with the aim of improving its discriminatory power, other VNTR loci were subsequently added, deleted or changed. These loci included MLVA20 by Le Flèche *et al*. [13], MLVA15 by Keim *et al*.[14–15], MLVA25 by Lista *et al*.[16] and all 31 VNTR markers (MLVA31) that were used to characterize *B*. *anthracis* genotypes in Namibia [17]. More recently, Thierry *et al*. [18] suggested that the MLVA7 scheme, which was based on seven loci (i.e., *vrrA*, *bams03*, *bams05*, *bams22*, *bams34*, *bams44*, and *vntr23*), were chromosomally located and employed as first-line assays. Although more data is required, the MLVA7 scheme took advantage of less loci and did not require precision DNA sequencing equipment. Thus, the MLVA7 scheme is a potentially useful and promising approach.

The canSNP are based on 13 rare single nucleotide polymorphisms that were selected from 1000 SNPs and originally identified on whole genome sequences of *B*.*anthracis*. Additionally, canSNP resolved 1033 isolates into three major lineages (i.e., A, B, and C), which were further sub-divided into 12 clonal sub-lineages. This system is often used to identify a broad genetic group. The SNRs are variable-number tandem repeats with very high mutation rates. The same MLVA genotypic strains can be distinguished by SNR markers. This approach was used as a molecular epidemiological tool for examinating the closely related isolates [19], and we believe it thus serves utility in tracking the geographical route of *B*.*anthracis*.

The presence of *B*.*anthracis* was recorded in historical Chinese medical texts in which anthrax was clearly presented in China for more than 5000 years [20]. Even today, anthrax remains a significant disease, especially in remote and under-developed villages and towns [21]. A group that worked on the surveillances of anthrax reported that there were 72 outbreaks in 10 provinces of China with 8998 human cases between 1990 and 1994. Additionally, another report showed that the average annual number of human cases was approximately 2115 between 1985 and 1994 [22]. However, there are a limited number of studies on anthrax with respect to North-East China.

From 2010 to 2014, our laboratory determined nine anthrax cases occurred in three provinces in North-East China (i.e., Jilin, Liaoning and Inner Mongolia). These cases were found in some of the local farmers and butchers that had contracted cutaneous anthrax during their occupational activities of butchering cattle and goats. These cattle and goats had died from anthrax or had been slaughtered according to the “Animal Epidemic Prevention Law of the People’s Republic of China (2007).”

In this current paper, we report and confirm the typing results of seven isolates that were recovered from these events. Furthermore, the genotypes of two *B*.*anthracis* vaccine strains, which are currently used in China, were determined. Finally, the possible causes of these incidences of anthrax in China are also discussed.

## Materials and Methods

### Ethics Statement

The study protocol was approved by the local Review Board of the Academy of Military Medical Sciences (AMMS). This study was carried out in strict accordance with the recommendations found in the guide for the care and use of tissue samples of the AMMS. One body fluid sample was collected from blister fluid of the arm, following provision by the patient of oral informed consent, due to the patient being illiterate. The oral consent was documented by the attending physician in the people's hospital of Inner Mongolia. This consent procedure was approved by the Review Board of the Academy of Military Medical Sciences.

Depending on the permission that was obtained from the owners of the farms, two beef, twenty-five OX-blood and five mutton samples were collected from the demised animals in eight farms in Jilin and Liaoning province, and none of the specimen collections involved either endangered or protected species. No specific permissions were required for these locations. Our laboratory was the designated unit to manage and deal with the identified anthrax cases by the Chinese Ministry of Agriculture. Thus, no permits were required for our collection of blood and tissue samples from the animals.

Sampling methods were supported by Anthrax in Humans and Animals- 4^th^ edition, as published by the WHO, OIE and FAO. This consent procedure was approved by the local Review Board of the Academy of Military Medical Sciences. All efforts were made to minimize suffering of the animals.

### Bacteria Isolation and Detection

In this paper, the meaning of strain refers to one colony that has been recovered from one anthrax case, albeit there were many isolates that could have been recovered from different animals or other types of specimens derived from any potential outbreak.

Samples were obtained from eight animal anthrax cases and one patient anthrax case. The nine anthrax sites were distributed in separate villages (S1 Table), with the exception of ZL-1 and ZL-2 strains. The vaccine strains known as Anthrax Spore Vaccine No. II Strain (also named C40-205) and Anthrax Spore Vaccine Non-capsulated Strain (also referred to as C40-202) were derived from the Lanzhou Institute of Biological Products. All samples were collected from 2010 to 2014 in Jilin, Liaoning and Inner Mongolia, three provinces that are located in the North-East of China.

With the exception of PCR detection, samples were analyzed in a biosafety containment level 3 mobile laboratory. Sampling methods and protocols for anthrax diagnostic tests and pathogen isolation were described in the OIE manual [23]. Briefly, blood or body fluid samples that had been collected from recently demised or infected animals which were inoculated directly on to blood agar plates. Then, before inoculation, frozen anthrax meat and soil samples from the animal carcass sites were grounded into pieces in two volumes of sterile distilled water and placed in a water bath at 62°C for 30–60 minutes. Following this procedure, all samples were incubated overnight at 37°C on PLET (Polymyxin, Lysozyme, EDTA, Thallous acetate) agar medium [24].

Before incubation, blood and meat samples were distributed as specimen smears on standard light microscope slides, and then fixed, and stained with Gram stain. Based on morphological observations of bacterial and colony appearance, suspected instances of *B*.*anthracis* were selected and identified by two confirmatory tests, which included the gamma phage (strain AP631, Lanzhou Institute of Biological Products. Co. Ltd) lysis assay and the penicillin (Sigma) susceptibility assay, both of which were conducted according to the OIE manual [23].

### DNA Preparation

DNA templates were prepared from 100 mL of an LB (Oxoid, UK) culture using a DNA extraction kit (QIAamp DNA Mini Kit, QIAGEN, Valencia, CA, USA) according to the manufacturer’s instructions. Before PCR detection, all prepared DNA templates were filtered through a 0.22 μm spin filter (Thermo Pierce, USA).

### Molecular Characterization

PCR were performed on an ABI GeneAmp 9700 (Applied Biosystems, USA) PCR amplification device with reagents that included Phusion high-fidelity DNA polymerase (New England Biolabs, Inc., Beijing, China). According to previously published studies [25, 26], *B*.*anthracis* toxin genes were detected by PCR assay using specific primers that targeted the protective antigen gene (*pag*) and the capsule gene (*cap*).

MLVA15 of *B*.*anthracis* for 15 loci and canSNP analysis that targeted 13 loci were performed as previously described [14]. SNR5 analysis was performed on the basis of methods that were previously developed by Stratilo *et al*. [19] and Kenefic *et al*. [27]; however, the SNR loci that were analyzed in this paper were modified to CL10, CL12, CL33, CL35 and CL60. The primer sequences were identical to those previously described [19], from which single PCR analyses were performed. The cycling parameters for all amplifications were 94°C, 2 min followed by 40 cycles of 94°C, 30 s; 45°C (CL12), 50°C (CL35), 55°C (CL10 and CL33), 60°C (CL60) for 30 s; and 72°C, 30 s; followed by a final elongation reaction of 72°C, for 10 min.

With the exception of canSNPs products that were directly sequenced with the amplification primers, other PCR products were cloned into pGEM-T Easy Vector (Promega, Biotech Co., Ltd., Beijing, China) and then sequenced with the forward primer SP6 and the reverse primer T7. Sequencing reactions were performed on the ABI 3730 XL automated sequencer (Applied Biosystems Inc.) by Comate Bioscience Co., Ltd, Jilin, China.

## Results

### Case Introductions

**Fig 1:**
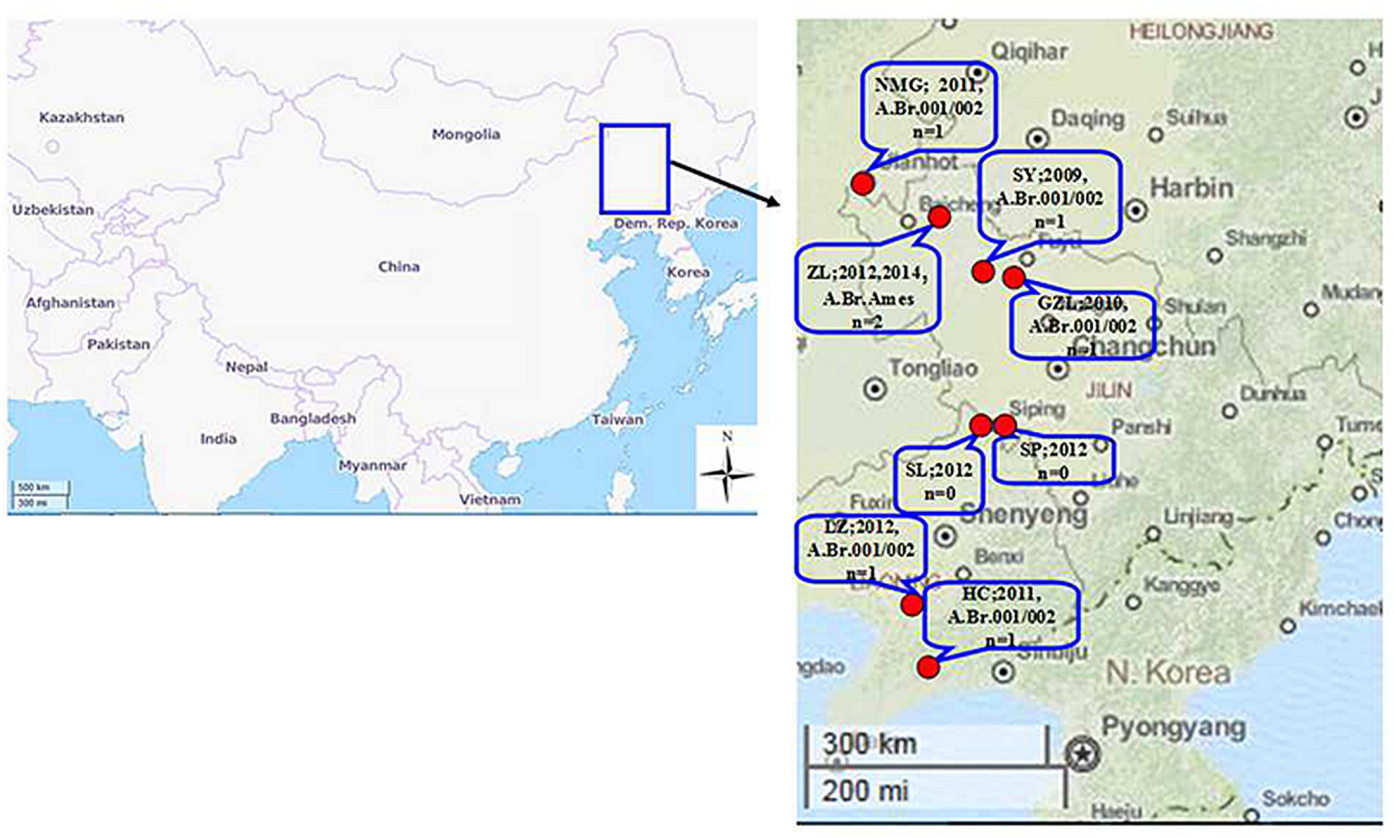
Geographical distribution of *B*. *anthracis* isolated in the North-East of China.

Nine anthrax cases occurred in North-East China (Fig 1, and S1 Table) during the summer or autumn of 2010–2014.

In these cases, 58 human individuals had contracted cutaneous anthrax but remained alive, 58 animals had died of anthrax, and 493 animals were slaughtered according to “the Animal Epidemic Prevention Law of the People’s Republic of China (2007)”. There were some common feature of these cases. For example, all patients had occupations that were either butchers or cooks with open wounds during butchering or cooking, and presented typical anthrax symptoms, as lesions of black eschar on the fingers or arms, developed over a period of five to seven days. In addition, butchers and cooks without open wounds or even those who had ingested the cooked anthrax infected meat did not contract anthrax.

Moreover, in each of these anthrax case, human anthrax cases were reported earlier than the animals cases. When officials traced the human cases, they just found the carcasses of the animals had been prepared for consumption by the patients. According to the descriptions provided by the local farmer, all of the animal cases were subacute, the animals became depressed, with weakness and prostration, and following their death, some cattle presented belly expanding to explosion, as well as hemorrhage of unclotted blood from the nose, mouth, vagina and/or anus. Further investigation showed that, although the time courses of these cases were similar, the status of the disease were irrelevant according to the location and the animal’s mobile routes, with the exception of the two cases outbroke in Baicheng city(2012, 2014).

Once the cases were confirmed, the control and prevention procedures were initiated. Animals that lived in the same stall were slaughtered and then burned, following which the carcasses were deeply buried and covered with lime and sprayed with surface disinfectant and set with a striking marker.

All animals within the village were injected with Anthrax Spore Vaccine No. II Strain or Anthrax Spore Vaccine Non-capsulated Strain. At the end of the vaccination procedure, a plan was drafted to monitor the location for three years. In addition, brochures that described anthrax, were distributed to farmers in the local villages.

### Bacteriological Characterization

Seven strains were recovered from samples of the nine anthrax cases. *B*. *anthracis* were readily isolated from blood or body fluids of a recently dead animal. The colony was relatively large with a diameter of approximately 0.3–0.5 cm, and was non-hemolytic and grey-white with a ground-glass, rough appearance. The vegetative cells of *B*. *anthracis* were large, ellipsoidal central spores that did not swell. The cells stained strongly Gram positive and appeared as long chains *in vitro*, and were paired or short chained *in vivo*, and they could be lysed by gamma phage and were sensitive to penicillin. These strains all harbored two toxin genes that were detected by conventional PCR assay. No *B*.*anthracis* strains were recovered from the infected meat samples brought by the farmer in Siping and Shuangliao provinces in 2012 because of the complication of heavily polluted soil contamination of the specimens. Both cases were confirmed by the presenting symptoms and positive confirmation of the presence of *pag* and *cap* genes by PCR assay. In addition, most of the non *B*. *anthracis* strains grew on the PLET agar, and were found to be members of the genus *Bacillus*, such as *B*.*cereus* and *B*.*mycoides*, confirmed by 16S rDNA sequencing (data not shown).

### Genotyping

**Fig 2:**
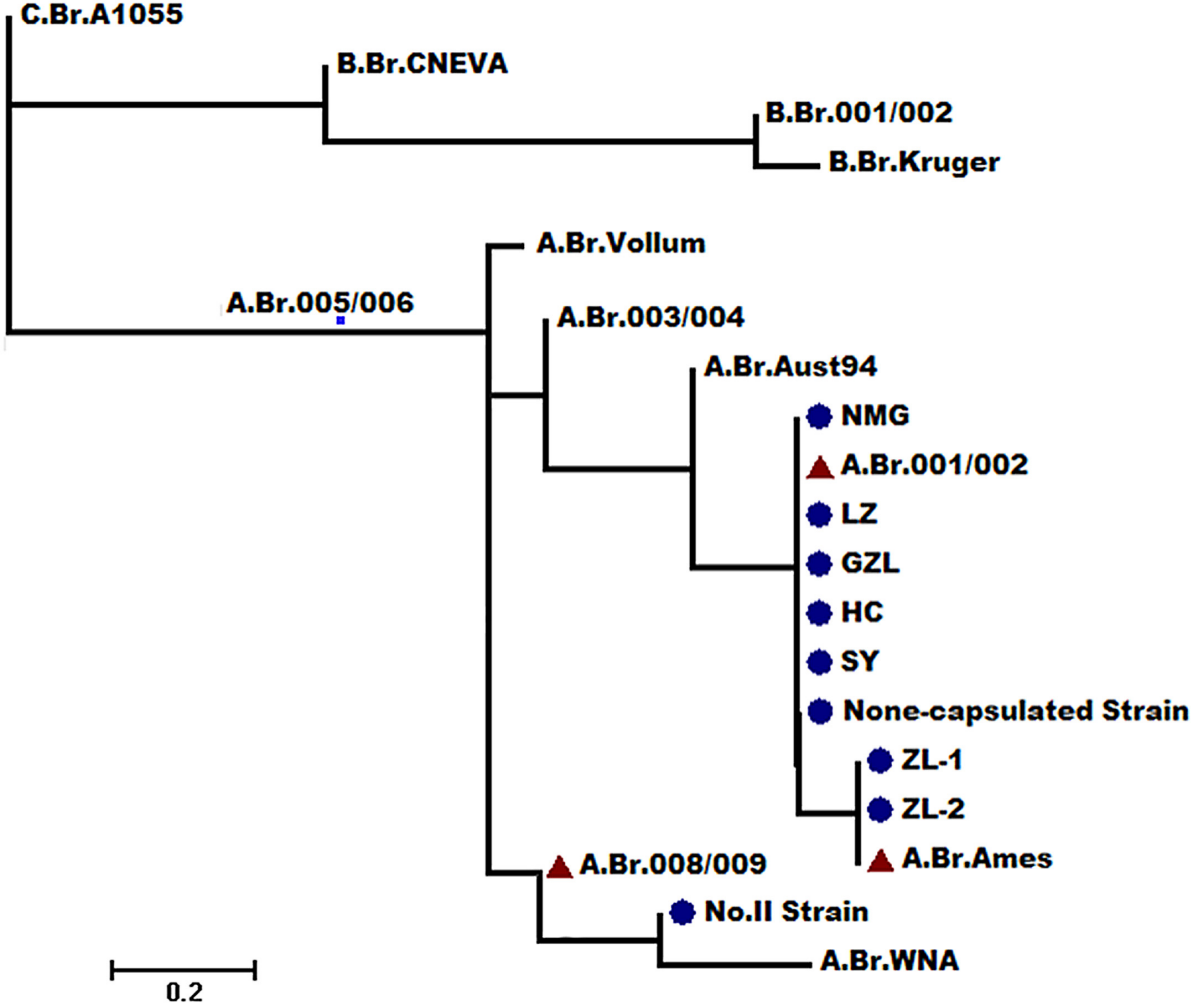
Phylogenetic location of the isolated strains and vaccine strains of the global mapping of the *B*.*anthracis* phylogenetic tree(14).

CanSNP analyses assigned five recovered strains and the Anthrax Spore Vaccine None-capsulated Strain to A.Br.001/002 sub-lineage, classified the remaining two recovered strains to A.B Ames subgroup, and classified the vaccine strain Anthrax Spore Vaccine No.II Strain to A.Br.008/009 (S2 Table and Fig 2). A.Br.001/002 and A.B Ames were both closely related, sharing a common ancestor, and these results corresponded to those of Simonson [28]. The A.Br.001/002 sub-lineage was a major presence in most regions of China, and 26 strains were distributed in Liaoning province and these were also of this sub-lineage. The two other strains (i.e., ZL-1 and the ZL-2 strain derived from Baicheng city) were canSNP A.Br.Ames, which was widely distributed in west of Inner Mongolia in China and USA, only some sporadic cases have been described in Europe [28]. This was also the first finding of the A.Br.Ames sub-lineage being present in North-East China. The vaccine strain Anthrax Spore Vaccine No.II Strain, which is popularly used in China, was A.Br.008/009, a sub-lineage that predominated throughout Europe, and was only found to be distributed in Xinjiang province, which is the most Western province in China [26].

MLVA15 analysis of the seven recovered strains showed closely related genotypes (S2 Table). Compared with Keim *et al*. [12], four genotypes were among the canSNP A.Br.001/002 sub-lineage, and identical genotypes among the canSNP A.Br.Ames sub-lineage. Anthrax Spore Vaccine Non-capsulated Strain was identical to the LZ strain, but the Anthrax Spore Vaccine No. II Strain was not precisely positioned in the MLVA8 scheme.

According to the results of SNR analysis (S2 Table), and with the exception of two strains derived from Baicheng city (i.e., ZL-1 (2012) and ZL-2 (2014), the other profiles were different from each other, which meant that only ZL-1 (2012) and ZL-2 (2014) were derived from the same clone.

## Discussion

An analysis of the causes of the nine anthrax cases showed that a lack of knowledge of the farmers was an important problem that had to be solved. The anthrax prevention and control system are completed in China, including laws and regulations, management organizational structure, infrastructure development, and report procedures with a diagnostic technique guidelines document, and an appointed laboratory. Moreover, animal anthrax cases should be regularly reported to the related office. However, in some under-developed villages, farmers are unaware of anthrax, and are ignorant of its biology and are even unaware that dead anthrax carcasses should not be prepared for human consumption. In some cases they would likely handle the carcasses covertly to reduce their financial losses. Clearly, this is a potentially serious problem, which can likely only be resolved by a strengthened system of community education programs and supervision activities, which would represent the most important steps designed to control the disease.

In China, free-ranging remains a common farming style in villages, but some farming regions are a naturally infectious area of anthrax outbreaks, land consequently live-stock were often infected by *B*. *anthracis* when being farmed, so many animal anthrax cases are known to be recurrent for anthrax outbreaks [29]. From the SNR analysis, both the ZL-1and ZL-2 strains were derived from the same clone, and further investigations showed that there was a big meadow on which to graze animals. In addition, farmers located around the meadow would herd their livestock there, which suffered from the issue of experiencing a pooling effect following periods of heavy rainfall during the wet season because of its low lying area. The ZL-1 strain was isolated from cattle that died from anthrax in that meadow, and the ZL-2 strain was isolated from a piece of cut meat that was obtained from a sheep carcass purchased from a peddler who could not be sought and identified. However, it was likely that the sheep once grazed there according to the best recollections of the local farmers. Although the time period between the two cases was about two years, and the meadow had been prohibited entry, repeat anthrax infections nonetheless occurred occasionally.

Anthrax surveillance at the location of the onset spot, was conducted every summer, which included epidemic studies and environmental sample (soil and water) detection. By this approach, no *B*.*anthracis* was detected, at least by the method of Stratio *et al*[30].

The genotypes of all of the recovered strains were common Chinese types, which indicated that they were indigenous Chinese strains in origin, and not foreignstrains that could have been introduced. The results of Simonson *et al*. [28] showed that the sub-lineage A.Br.Ames “has a major presence in most of China”,which was based on only four strains that were derived from Western Inner Mongolia. However, our results provided supporting data to that conclusion. Clearly, more strains are needed from other regions, especially from Central and Southern China, because the numbers of cattle in China are very large.

After the discovery of the anthrax vaccine by Pasteur in 1881, many vaccine strains were used to prevent animals contracting anthrax worldwide [31]. From previous results, Pasteur I (ATCC 4229, strain 75.16) was pXO1^-^/pXO2^+^ [32], was grouped into the new A.Br sub-lineage [33]. In addition, Pasteur II (strain 74.12) was pXO1^+^/pXO2^+^[33], was grouped into A.Br.008/009 without MLVA and SNRs information [33]. The most widely used animal anthrax vaccine was developed by Max Sterne in 1937, which was a live, non-encapsulated strain. However, today there are many different Sterne strains being used in different laboratories. For example, strain 77.2 was pXO1^+^/pXO2^-^ non-capsulated that was grouped into A.Br.001/002 [33,34]; the strain used in the MLVA8 by Keim *et al*. was pXO1^+^/pXO2^+^ and was grouped into A3.b, genotype 59 and 61 [31]. However, a pXO1^+^/pXO2^-^ strain had been grouped into the MLVA8 A3.b genotype 63 by Stratilo *et al*. [19].

For several decades, the Anthrax Spore Vaccine No. II Strain, and the Anthrax Spore Vaccine non-capsulated Strain were the only approved vaccines for widespread use in continental China. However, it is currently not precisely known when and where the strains were introduced, and the biological and molecular characterization of those strains has not been determined until now. From our results, the molecular character of the Anthrax Spore Vaccine non-capsulated Strain, pXO1^+^/pXO2^-^, A.Br.001/002 in canSNP, A3.b genotype 63 in MLVA8, was similar to the strain used in Stratilo’s experiment. Although the SNR result showed that they were not derived from the same clone [19], it is formally possible that they might be derived from the same ancestor. In the case of the Anthrax Spore Vaccine No. II Strain, pXO1^+^/pXO2^+^, A.Br.008/009 in canSNPs is not positioned in MLVA8, but resembled that from the Pasteur II lineage [33]. We also sequenced the *bclA* [35] gene of the vaccine strains, but in this analysis, no similar sequences were found when blasting the data (data not shown). More detailed investigations should be conducted, and include biological and molecular characterization. We submit that our results are beneficial in distinguishing a recovered strain from a vaccine strain of *B*.*anthracis*. In addition, we have provided information that should enable more detailed research.

In summary, the genotypes of the recovered strains of *B*.*anthracis* and of the approved vaccines were determined. It was shown that the sub-lineage A.Br.001/002 was dominant like that in other provinces. A.Br.Ames was first detected in this region and the molecular characteristics of the vaccines did not correspond to any of the other previously published strains. Popularization of the knowledge and regulations concerning the recognition, handling and treatment of anthrax outbreaks in remote villages can assist in prevention and control strategies of the events that typically leading to the onset of, or contracting anthrax. It is recommended that the Anthrax Spore Vaccine Non-capsulated Strain is the only vaccine used in the whole of China when one considers the toxicity of anthrax outbreaks and the genotype.

## Supporting Information

S1 Table Anthrax in three province in North-East China 2010–2014. * Strains were isolated from animals except as mentioned; (a) from a human patient; (—) no data available; (#) PCR positive but failed isolation from contaminated meat.
(XLS)

S2 Table Genotyping of the isolated strains and vaccine strains (14). (XLS)
